# The Impact of Spray Cryotherapy on Lesion-Induced Osteitis in a New Murine Experimental Model

**DOI:** 10.3390/medicina59050897

**Published:** 2023-05-07

**Authors:** Ioana Maria Porfire (Irimia), Alexandru Flaviu Tabaran, Madalina Luciana Gherman, Veronica Elena Trombitas, Silviu Albu

**Affiliations:** 1 IInd Department of Otorhinolaryngology, ‘Iuliu Hatieganu’ University of Medicine and Pharmacy, 400015 Cluj-Napoca, Romaniasilviualbu63@gmail.com (S.A.); 2 Department of Anatomic Pathology, University of Agricultural Science and Veterinary Medicine, 400372 Cluj-Napoca, Romania; alexandru.tabaran@usamvcluj.ro; 3 Experimental Centre, University of Medicine and Pharmacy ‘Iuliu Hatieganu’, 400349 Cluj-Napoca, Romania

**Keywords:** chronic rhinosinusitis, murine experimental model, low pressure cryotherapy, osteitis

## Abstract

*Background and Objectives*: Endoscopic sinus surgery is considered the gold management strategy for difficult-to-treat chronic rhinosinusitis. The inflammatory bony process is incriminated as being involved in the unfavorable evolution and recurrence of the disease. Osteitis is significantly increased in patients that have been previously submitted to surgery, and it is more often present in patients with extended radiological disease and in patients undergoing revision surgery. The aim of the research is to demonstrate the presence of inflammations and neo-osteogenesis associated with nasal mucosal surgical injury and the correlation between their severity and to evaluate the efficacy of low-pressure spray cryotherapy in reducing inflammation and bone remodeling. *Materials and Methods*: The experimental murine model was conducted over a period of 80 days and included a total of 60 adult female Wistar rats, with three periods of withdrawal of 20 individuals each from the experiment. After inducing a bilateral mechanical injury by brushing, low-pressure spray cryotherapy application was performed unilaterally, and tissue samples were prepared specifically for histological analysis. The scores for inflammation and osteitis were compared over time and between the two nasal fossae. *Results*: Osteitis and inflammation were induced by a simple mucosal brushing lesion, similar to surgical injury. We identified the presence of inflammation in 95% of the specimens, and it was present over time. Moreover, criteria for bone remodeling were clearly highlighted in a percentage of 72% of the specimens. There was a direct correlation between the severity of inflammation and neo-osteogenesis, with a statistical significance of *p* = 0.050. Low-pressure spray cryotherapy was safe and effective in reducing inflammation (*p* = 0.020) and osteitis (*p* = 0.000) with a safety profile. *Conclusions*: Low-pressure cryotherapy reduces the severity of mucosal inflammation and osteitis in lesion-induced neo-osteogenesis.

## 1. Introduction

Although endoscopic sinus surgery (ESS) is traditionally the standard of care for chronic rhinosinusitis (CRS), complications still occur [1]. Recurrent disease due to excessive scar formation, adhesions and sinus osteo-meatal stenosis require revision surgery; thus, the improvement of therapeutical methods remains a constant quest [2]. Spray cryotherapy safety, efficacy and tolerability have been investigated in a wide array of medical domains. In otorhinolaryngology, this therapy was studied with a focus on rhinologic outcomes, revision surgery, quality of life and concurrent medication [3,4]. Even so, the results are encouraging, and long-term observation and careful histological examination may be required. On a solid experimental basis, additional clinical trials on the use of cryotherapy with endoscopic sinus surgery should be attempted [5].

Low-pressure spray cryotherapy works on the superficial layer of the mucosa and produces instant tissue freezing and cell death, with minimal local side effects. Ever since, the basic features of cryosurgical techniques, such as rapid freezing, slow thawing and repetition of the freeze–thaw cycle, were established. The nature of the cryosurgical injury has been the subject of numerous investigations in an effort to define the appropriate temperature–time dosimetry of the freeze–thaw cycles of cryosurgery to improve the delivery method and the monitoring techniques [6]. Cryotherapy, a tool for dermatologists and gastroenterologists for many years, has received increasing attention among otolaryngologists. The usefulness of this method has been demonstrated in experimental animal studies in maintaining maxillary antro-meatal and myringotomy permeability in rat and rabbit experimental models [2,5,7,8,9]. It was demonstrated as safe, well-tolerated and efficacious in endoscopical application to the middle meatus used to freeze the posterior nasal nerve in chronic rhinitis. In the esophagus [3,4] even more, it could have a considerable benefit in the difficult-to-treat CRSwNP management by its effect on microbial biofilms [8].

The first randomized prospective controlled study of cryotherapy as an alternative treatment for chronic rhinitis conducted by Del Signore et al. [10] demonstrated higher ranking scores for nasal symptoms and quality of life in cryo-treated patients. Kompelli et al. [11] validated cryotherapy as a safe and efficient treatment option for chronic rhinitis by performing a systematic review exploring the utility, efficacy, safety and durability of treatment responses. A significant improvement in nasal obstruction, rhinorrhea and recurrent upper respiratory infections were reported. Additionally, the few reported complications such as epistaxis, nasal obstruction and crusting demonstrate the safety of the method.

On a molecular level, bony remodeling is promoted by several factors. Chronic inflammatory disease and cytokine expression, such as prostaglandins, leukotrienes and growth factors, are continuously occurring processes. Osteoblast and osteoclast activation is regulated by members of the transforming growth factor family TGF-β. Additionally, inflammatory cytokines outside of the TGF-β superfamily may also be involved in bone remodeling. [12] IL-1 β expression was associated with fibroblast and osteoblast stimulation and interleukin (IL)-6, IL-11 and TNF-α gene expression were noted in the bone tissue. An upregulation of bone morphogenetic protein (BMP) such as BMP8 and BMP9 in the sino-nasal tissues has been observed [12,13,14].

In this regard, our research strengthens the idea that osteitis and inflammation may be induced by simple mucosal brushing lesions, resembling surgical injury. Moreover, we assessed the correlation between the severity of inflammation and neo-osteogenesis by employing a grading scale for inflammation and osteitis. We also evaluated the efficacy of low-pressure spray cryotherapy in reducing inflammation and neo-osteogenesis.

## 2. Materials and Methods

### 2.1. Animal Preparation

The experimental protocol was approved by the Institutional Veterinary Ethics Committee (23/4 November 2020) and was conducted according to the Declaration of Helsinki, legal regulations and international guidelines on animal experimentation.

We conducted our experiment over a period of 80 days. We studied a batch of 60 female Wistar rats, aged 16 weeks, weighing 300.00 g (±17.965 g standard deviation). Animals were housed and cared for according to standard rules under controlled conditions: temperature 22 ± 2 °C, humidity 50 ± 10%, light–dark cycle 12–12 h, with access to tap water and solid food ad libitum.

Using narinoscopy, all specimens included in the study had normal-appearing nasal mucosa without mucous or mucopurulent nasal secretions; thus, we concluded that the inflammation was procedurally induced, and possible pre-existing inflammation was ruled out.

**Figure 1:**
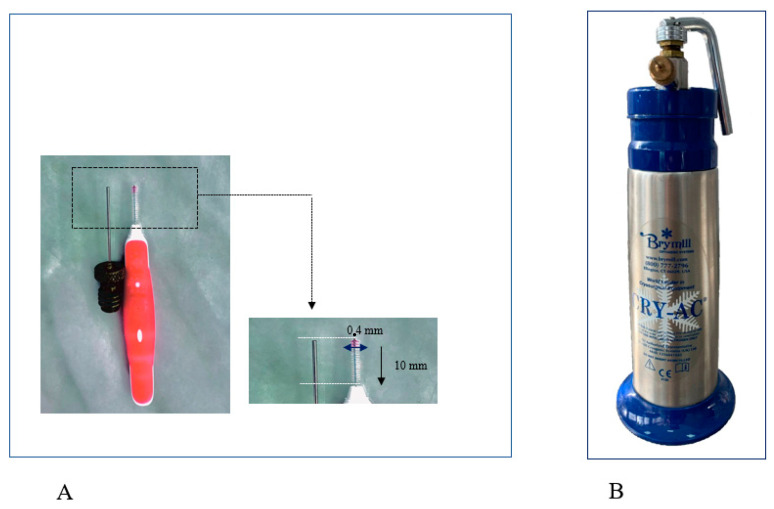
(**A**) The interdental brush with a radius of 0.4 mm and a length of 10 mm, used to produce superficial lesion of the nasal mucosa; (**B**) cryotherapy cannula for liquid nitrogen (cryotherapy). The Brymill CRY-AC-3 CryoPro Cryogenic System (Williams Medical Supplies Ltd., Rhymney, UK), a device that provides uniform and wide distribution of liquid nitrogen −196 °C, was used during the study for spray cryotherapy.

After prior weighing and numbering, anesthesia was performed by intraperitoneal injection of Ketamine 80 mg/kg (0. 04 mL Ketamine-Vetased) and Xylazine 8 mg. Following the protocol described in our previous pilot study [15], a mechanical lesion of the nasal mucosa was induced, bilaterally. We performed three rotations, clockwise, using a 0.4 mm radius brush (Figure 1A).

**Figure 2:**
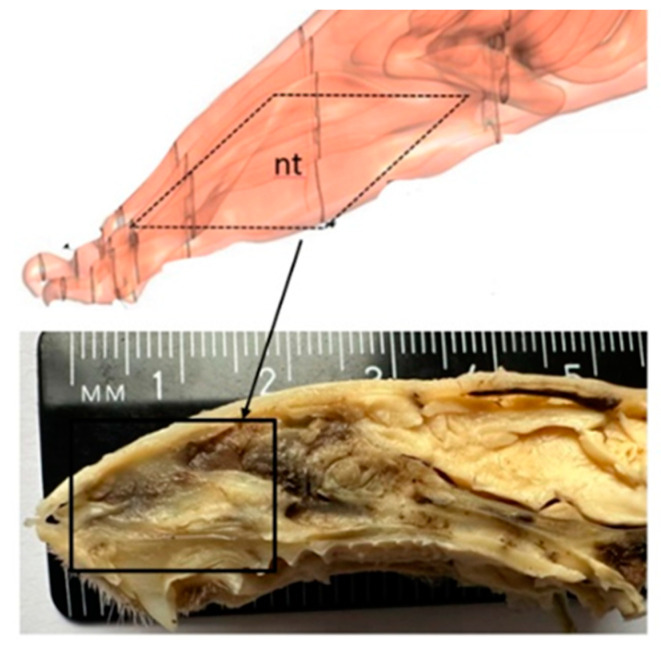
Rat nasal anatomy, sagittal section (nt = nasal turbinates).

To produce similar lesions in all subjects, we controlled the size and depth of the lesion using the same induction procedure and identical brushes. A 16-week-old rat weighing about 300 g has a nasal fossa length of 9.1 mm (±0.3 mm standard deviation), measured between an anterior point, represented by the posterior surface of the upper incisor, and a posterior point, defined as the most anterior section with an incomplete septum. The anterior plane closest to the nostrils is followed by the nasal passages, where the nasal turbinates create a curved relief and a large surface [16,17] (Figure 2).

After the first injury, every individual was submitted to unilateral low-pressure cryotherapy spray at the level of the right nostril in two clicks of 1 s each with 35 s pause between applications to allow complete thawing of the tissues. Superficial cryotherapy was applied using CryoPro cryotherapy system (Williams Medical Supplies Ltd., Craiglas House, The Maerdy Industrial Estate, Rhymney NP22 5PY, UK), a device that provides uniformity and width of liquid nitrogen distribution at −196 °C (Figure 1B). Precise handling of the device allowed liquid nitrogen to be sprayed for 1 s on the circumference of the injured mucosal area. After application, 35 s was allocated for thawing the sprayed area, after the second cycle of 2 s was applied. The maneuver was immediately followed by the appearance of bilateral epistaxis, being more abundant on the cryotherapy induction side. In all cases, the epistaxis was stopped spontaneously, and other than this, no other adverse events were observed. Contralaterally, the left nasal fossa was considered a control.

### 2.2. Study Protocol

The animals were withdrawn from the experiment, 20 individuals each at different periods, at 14, 30 and 80 days, and euthanized by intramuscular administration of an anesthetic overdose of Ketamine (Vetased). Then, the skulls were harvested.

**Figure 3:**
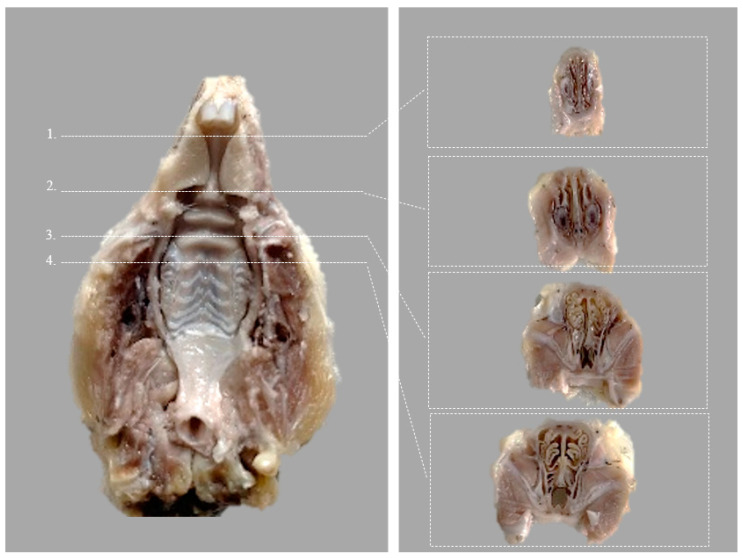
Ventral view of the rat hard palate after harvesting, indicating the four coronal sections (Modified after (Renne et al., 2009)) [19]. Corresponding transverse incisions are at the level of the: 1. posterior part of upper incisor teeth; 2. incisive papilla; 3. second palatal ridge; 4. first upper molar teeth.

Tissue samples consisting of rat heads (bone and adjacent soft tissues) were prepared for histological analysis. After fixation in 10% neutral-buffered formalin (7–14 days), the samples were decalcified in Osteorall L (decalcifier for small anatomical parts, RAL DIAGNISTICS, Martillac, France). After a previous pre-testing performed on a similar tissue sample and good quality histopathological results, Osteorall L solution was used. Osteorall L replaced the decalcification solution consisting of 8% HCl and 8% H2CO2 in a 1/1 volume, described by Prophet E. B. et al. [18] and used in our pilot study. After complete decalcification at 7 days, the soft tissues and the lower jaw were removed and the splanchnocranium was cut into standard serial coronal sections, according to the scheme below (Figure 3).

The standard histological cassettes containing coronal slices were positioned rostral face down and embedded in paraffin wax. The samples were washed in tap water for 30 min, subjected to graduate chemical dehydration in ascending baths of ethanol (70%, 90%, 95% and 100%), cleared with 100% Xylene and embedded in high-melting temperature paraffin wax. Paraffin blocks were chilled at −4 °C, and seriate tissue sections were cut at 3 μm with a rotary microtome.

### 2.3. Histological Analysis

Histological analysis was performed by a single unblinded pathologist using different high-powered fields with an Olympus BX51 microscope model.

From a histological point of view, the presence of inflammation and osteitis criteria and the differences between the two nasal fossae—the right cryo-treated fossae and the control left nasal fossae—were evaluated in terms of general morphology.

We used Mayer’s hematoxylin-eosin staining and followed the identification and quantification of the criteria of inflammation, respectively, of osteitis, as previously defined by De Campos et al. [20].

Thus, we followed the diffuse purulent catarrh, mononuclear infiltrate, the presence of edema, the presence or absence of epithelial hyperplasia and exudate, inflammatory periosteal thickening, the presence of activated osteoblasts, osteoblastic rimming with or without osteoid deposition and the presence of osteoclasts.

### 2.4. Statistical Analysis

The analogy between the groups was conducted with a Chi-Square non-parametric test. The statistical analysis was performed, and a *p* value < 0.05 was considered significant. We further analysed the relationship between groups. The correlations between osteitis and inflammation, localization and their time relation were explored. In case of criteria with *p*-value at the common significance threshold of 0.05, we used post hoc cross-tabulations with adjusted residual and probability values, following the application of the Bonferroni correction.

## 3. Results

### 3.1. Histological Variations in Relation to Time

In the present experimental model, we carefully searched for the included criteria and the grading of mucosal inflammation and osteitis according to De Campos et al. [20], as mentioned and depicted above in Table 1. Subsequently, this grading system brought significant differences between actual results and those from the previously conducted pilot study by the additional quantification of grade 1 of inflammation and osteitis. The actual results are 95% vs. 64.7% for mucosal inflammation presence and 72% vs. 35.3% for osteitis identification, compared with our previous pilot research [15].

We identified the presence of inflammation criteria in 95% of specimens, unilateral or bilateral, graded as mild, moderate and severe.

At the level of the nasal meatus, we identified the presence of diffuse purulent catarrh in the meatus. Inflammatory infiltrate with polymorphonuclear cells was diffusely present, unilaterally or bilaterally, at the level of the septum and maxillary turbinates. Our analysis revealed that histopathological features, in simple light microscopy, were consistent with mucosal inflammation in 57 out of the 60 included, representing a percentage of 95%, of the specimens in varying grades, as follows: mild (45.6%), moderate (35.1%) and severe (21%). Histopathological findings were conclusive for inflammatory catarrh in the meatus. Mucosa inflammation with edema in lamina propria and mononuclear cells was diffusely present, unilaterally or bilaterally, at the level of the septum and maxillary turbinates. The nasal meatus was partly occupied by exudate, admixed with mucus and some necrotic cell debris (purulent catarrh). In the mucosal lamina propria edema, lymphocytes admixed with neutrophils and plasma cells were depicted. Squamous mucosal metaplasia with ulcerations of the mucosa and its polypoid hyperplasia, most likely in a postlesional context, was also highlighted.

We found criteria for neo-osteogenesis in 43 out of the 60 included, representing a percentage of 72% of the specimens, with the following percentages: 74% mild, 12% moderate and 14% severe. Bony remodeling was depicted at nasal turbinate structures, thickened with irregular extension. Within the trabecular bone of the nasal turbinate, there was periosteal proliferation with intense osteoblastic activation and proliferation, from osteoblast strings that start to produce bone tissue, to osteoblasts surrounded by osteoid matrix occasional osteoclastic. Bony resorption was suggested by the active osteoclasts in resorption lacunae and multifocal, prominent osteoid deposition, woven and trabecular bone, with variable degrees of mineralization and neo-osteogenesis with lamellar bone deposition.

The above-mentioned changes in terms of inflammation and bone remodeling have a zonal character, with propensity for the anterior-most sections of the nasal cavity, thus supporting the theory that inflammation processes can disseminate through bone architecture, disregarding the mucosal inflammation.

As well, the criteria for bone remodeling were clearly highlighted in 72% of the specimens. Within the trabecular bone of the nasal turbinates, periosteal proliferation with prominent osteoblastic activation and proliferation was clearly described. Occasional osteoclastic bone resorption and multifocal, prominent osteoid deposition with variable degrees of mineralization was highlighted.

### 3.2. Variable Differences in Relation to Time

**Figure 4:**
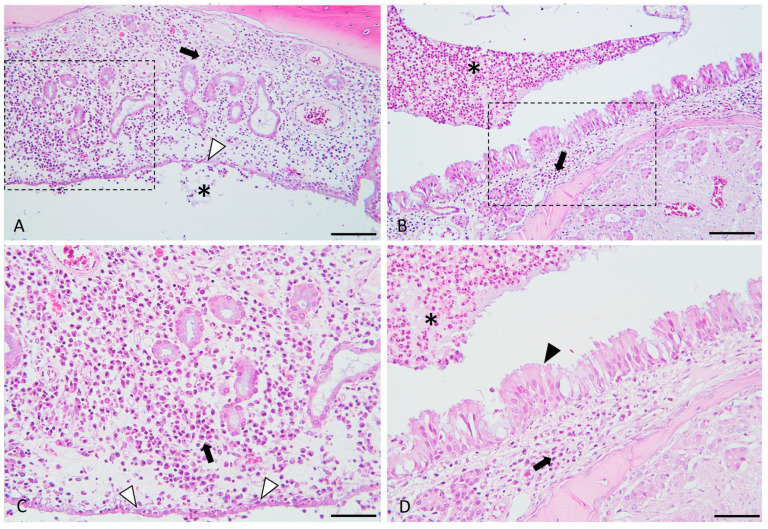
Histological micrographs presenting the key features of experimentally induced chronic rhinosinusitis. Images (**A**,**C**) (detailed area of image (**A**)): Diffusely, the lamina propria is distended, and the mucosal glandular elements are separated by many plasma cells and lymphocytes (indicated by the black arrow), admixed with edema. The epithelium is flattened, focally eroded and multifocally infiltrated (indicated by the white arrowheads) by the above-described inflammatory cells. The epithelium is focally covered by few neutrophils admixed with mucus and necrotic cell debris (indicated by the asterisk). Images (**B**,**D**) (detailed area of image (**B**)): Diffusely, the lamina propria is infiltrated by a moderate number of lymphocytes and plasma cells. The adjacent respiratory epithelium is multifocally hyperplastic (indicated by the black arrowhead) and covered by many neutrophils (viable and degenerated), admixed with mucus and a few necrotic cell debris (purulent catarrh).(indicated by the asterisks). H&E stain, ob × 20 (images (**A**,**B**), scale bar = 100 µm) and ob × 40 (images (**C**,**D**), scale bar = 50 µm).

**Figure 5:**
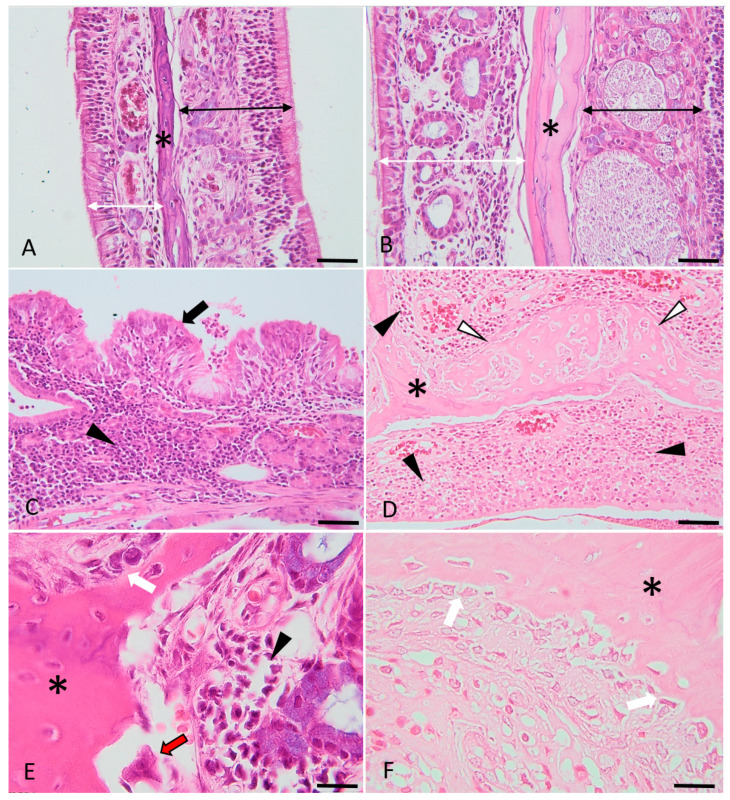
Histological micrographs presenting the key features of bone changes observed in experimentally induced chronic rhinosinusitis (images (**C**–**F**)), compared with the control (images (**A**,**B**)). Images (**A**,**B**): control images of the nasal turbinates, presenting the respiratory (indicated by the double white arrowheads) and sensory mucosa (indicated by the double black arrowheads) and the trabecular bone (asterisk). Images (**C**,**D**): Diffusely, the lamina propria is infiltrated by lymphocytes, plasma cells and macrophages (indicated by the black arrowheads). The respiratory epithelium is multifocally hyperplastic (indicated by the black arrows) or ulcerated (image (**D**)) and covered by a purulent catarrh. Within the trabecular bone of the nasal turbinates (indicated by the asterisks) (images (**D**–**F**)), there is periosteal proliferation with prominent osteoblastic activation and proliferation (images (**E**,**F**), white arrows), occasional osteoclastic bone resorption (image (**E**), red arrow) and multifocal, prominent osteoid deposition (woven and trabecular bone, with variable degrees of mineralization) (image (**D**), white arrowheads). H&E stain, ob × 20 (images A barr = 100 µm), ob × 40 (images (**B**–**D**), scale barr = 50 µm) and ×100 (images (**E**,**F**), scale barr = 20 µm).

The osteitis and inflammation histopathological parameters are highlighted above in Figure 4 and Figure 5.

The descriptive statistic of the variables used is summed up in the bellow subheadings.

To analyze the statistical variance between groups, we used the Chi-Square non-parametric test, observable in the tables bellow. For the *p*-value at the common significance threshold of 0.05, we applied post hoc cross-tabulations with adjusted residual and probability values and marginal corrections (Bonferroni-corrected threshold).

#### 3.2.1. The Presence and Evolution of Mucosal Inflammation over Time

**Figure 6:**
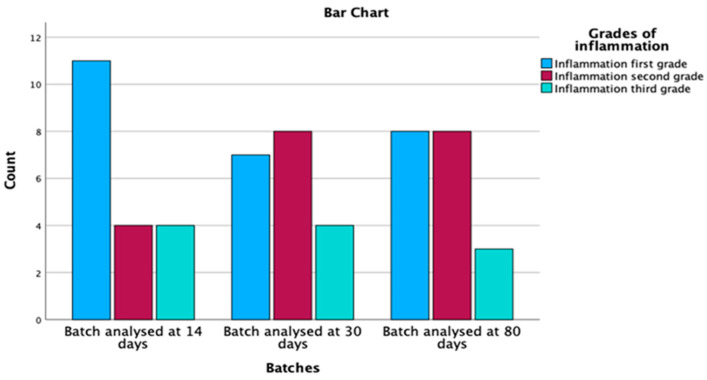
Inflammation distribution by batches and grades.

Out of the 60 specimens evaluated, 57 rats, representing a percentage of 95% out of the total, had signs of mucosal inflammation. Grade 1 inflammation was identified in 26 individuals (45.6%), grade 2 in 20 rats (35.1%) and grade 3 in 11 specimens (19.3%) (Table 2). Therefore, inflammation was present over time in a statistically significant manner (*p* = 0.050), represented in Figure 6.

As an evolution of the mucosal inflammation over time, we found maintenance of the total inflammation in all three groups, respectively, at day 14, 30 and 80, with an increase in grade 2 inflammation over time and a decrease in grade 1 inflammation while the number of those with grade 3 inflammation remained stationary.

#### 3.2.2. The Presence and Evolution of Bony Remodeling over Time

**Figure 7:**
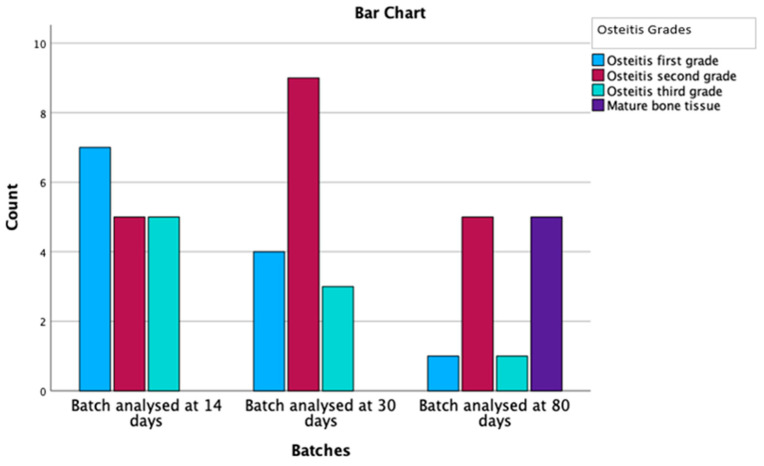
Osteitis grading and mature bone deposition distribution by batches.

Out of the 60 specimens evaluated, 43 rats, representing a percentage of 72%, had signs of bony changes. Grade 1 osteitis was identified in 14 individuals (32.6%), grade 2 in 20 rats (46.5%), and grade 3 in 9 specimens (20.9%) (Table 3). Therefore, bone remodeling was present over time in a statistically significant manner (*p* = 0.120), represented in Figure 7.

In regard to the variation in time of the osteitis grade, the number of those with osteitis decreased over time, so that in the 80-day batch, we had 10 vs. 17 individuals compared to the 14-day batch; a decrease of 41%. Additionally, of great importance is the presence of mature bone, which appears with a similar incidence as the one with which the osteitis decreases. Mature lamellar bone deposition has been reported exclusively in the 80-day batch in 50% of the subjects with osteitis. Deposition of lamellar bone has not been previously stated in the literature, probably since, prior to our study, no other experimental model had such a long follow-up period.

#### 3.2.3. The Positive Correlation between the Severity of Mucosal Inflammation and Bony Remodeling

The non-parametric Chi-Square for the association between inflammation grades and osteitis yielded a value of 6.000 with 2 degrees of freedom (df) and an asymptotic significance (*p*-value) of 0.050. Given that the *p*-value is exactly at the common significance threshold of 0.05, this result implies a marginally significant relationship between the grades of inflammation and osteitis.

**Figure 8:**
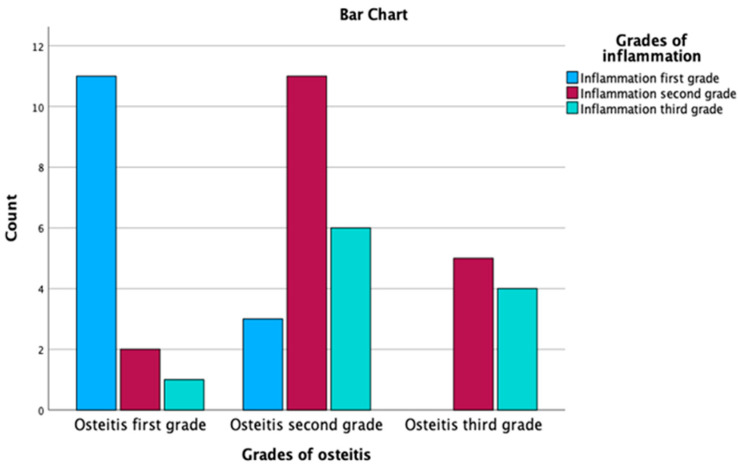
Correlation between the grade of inflammation and the grade of osteitis.

Regarding the test results for the association between grades of osteitis and grades of inflammation, a Chi-Square value of 4.233 was observed with 2 degrees of freedom and an asymptotic significance of 0.120. As the *p*-value exceeds the common significance threshold of 0.05, this result suggests that there is no statistically significant relationship between the grades of osteitis and grades of inflammation. Table 4 and Figure 8 present the relationship between inflammation grades and osteitis grades, along with adjusted residuals and probability values after applying the Bonferroni correction, with a threshold *p*-value of 0.0055. In the osteitis first grade, inflammation first grade is predominant with 78.6% of the cases within this grade, and this association is statistically significant, with a probability value of 0.000, which is below the threshold of 0.0055. The second and third grades of inflammation, with probability values of 0.011 and 0.054, respectively, are not statistically significant at the adjusted threshold.

In the osteitis second grade, none of the relationships between inflammation grades and osteitis grades are statistically significant, given their probability values (0.022, 0.103 and 0.536) are all above the threshold of 0.0055. However, it is still notable that the second grade of inflammation is the most frequent (55.0%) in this grade.

In the osteitis third grade, none of the relationships are statistically significant, as their probability values (0.019, 0.349 and 0.145) are above the threshold of 0.0055. Despite not being significant, inflammation second grade is the most prevalent in this grade, with 55.6% of the cases, and the third grade is also observed in 44.4% of the cases. Using the Bonferroni-corrected threshold of 0.0055, the table demonstrates that only the relationship between osteitis first grade and inflammation first grade is statistically significant. However, there are observable trends that higher inflammation grades are more prevalent in the later grades of osteitis.

#### 3.2.4. Efficacy of Low-Pressure Spray Cryotherapy in the Injury of Lesion-Induced Inflammation and Osteitis

**Figure 9:**
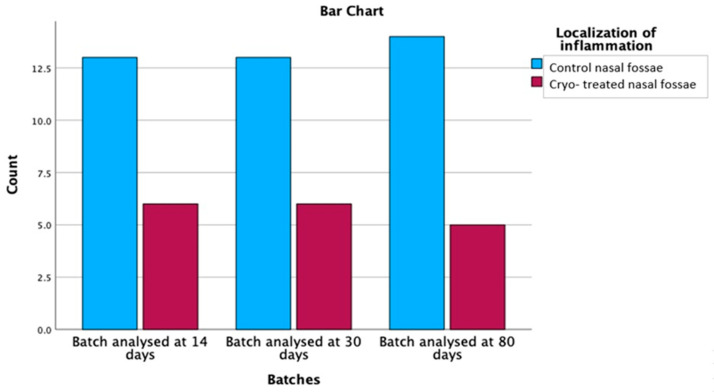
Comparison of inflammation distribution between control and cryo-treated, demonstrating its higher presence at the level of the control side.

Out of the 57 rats with mucosal inflammation, we identified histopathological changes at the level of the control nasal fossae in 40 subjects, representing a percentage of 70.2%. In only 17 cryo-treated fossae, hallmarks of inflammation were recorded (29.8%), as shown in Table 5. Comparing the mucosal inflammatory parameters in cryo versus control nasal fossae, the cryotherapy protective effect on inflammation was demonstrated with statistical significance (*p* = 0.002), graphically represented in Figure 9.

The relationship between inflammation localization and batches was found to be statistically significant (χ^2^ = 9.281, df = 1, *p* = 0.002). The observed count for control nasal fossae was 40, which was higher than the expected count of 28.5, with a residual of 11.5. On the other hand, the observed count for control and cryo-treated nasal fossae was 17, which was lower than the expected count of 28.5, with a residual of −11.5. These results suggest that there is a significant association between the inflammation localization and the analysed batches. Specifically, the control nasal fossae were more prone to inflammation, while the control and cryo-treated nasal fossae showed a reduced prevalence of inflammation across the three analysed batches.

We further analysed the relationship between batches, and inflammation localization was explored using post hoc cross-tabulations with adjusted residual and probability values, following the application of the Bonferroni correction (with a *p*-value of 0.0083). Batches included data analysed at 14 days, 30 days and 80 days, while inflammation localization was categorized into control nasal fossae and control and cryo-treated nasal fossae (Table 5).

After the Bonferroni correction, no significant differences were found between the batches and inflammation localization (*p* > 0.0083). These findings suggest that there is no significant association between inflammation localization and the analysed batches after applying the Bonferroni correction. Thus, the distribution of inflammation in control nasal fossae and control and cryo-treated nasal fossae appears to be similar across the three analysed batches.

**Figure 10:**
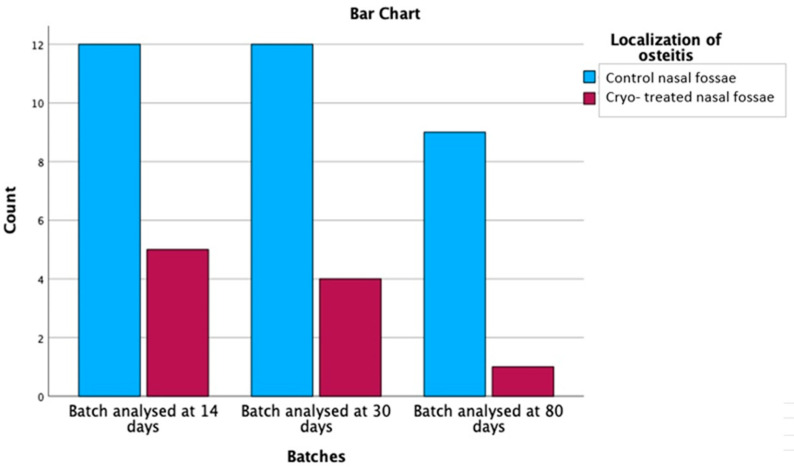
Comparison of osteitis distribution between control and cryo-treated side.

Additionally, out of the 43 rats with osteitis, 33 subjects (76.7%) had histological changes at the level of the control nasal fossae. In only 10 cryo-treated fossae, bone remodeling was reported (23.3%). Comparing bone changes between the control and the cryo-treated nasal fossae, the positive effect of superficial cryotherapy on bony changes was demonstrated, with statistical significance (*p* = 0.000), as graphically represented in Figure 10.

Focusing on the localization of osteitis, the non-parametric Chi-Square test yielded a value of 12.302 with 1 degree of freedom and an asymptotic significance of 0.000, suggesting a statistically significant relationship between the control nasal fossae and the control and cryo-treated nasal fossae in terms of osteitis localization.

The relationship between batches and osteitis localization was further examined by considering the cross tabs with adjusted residuals and probability values after applying the Bonferroni correction (*p*-value set at 0.0083). Table 6 depicts data from three different batches (analysed at 14 days, 30 days and 80 days) and the localization of osteitis in control nasal fossae and control and cryo-treated nasal fossae.

For the batch analysed at 14 days, there was no statistically significant relationship between the localization of osteitis, as the probability values of 0.440 for control nasal fossae and 0.440 for control and cryo-treated nasal fossae both exceeded the Bonferroni-corrected ***p***-value threshold of 0.0083. Similarly, no significant relationships were observed for the batches analysed at 30 days and 80 days, with probability values of 0.835 and 0.257, respectively, for both localization categories. These results suggest that the relationship between inflammation grades and osteitis grades did not vary significantly across the three batches analysed in this study. The lack of significant relationships across all batches supports the notion that the factors contributing to the development of osteitis might not be dependent on the timing of the batch analysis.

## 4. Discussion

Today, we have good evidence of mucosal changes in chronic rhinosinusitis, but very few recent investigations on bony changes (Giacchi et al., 2001 [21]). However, the molecular mechanism and functional significance remain unclear. Khalmuratrova et al., 2021 [22] highlighted the critical role that overlying inflammatory sino-nasal mucosa plays in the initiation of inflammatory processes at the bony level in patients with chronic rhinosinusitis. Due to the passage of inflammatory mediators through the Haversian canal system to non-adjacent bone structures, bone involvement may contribute to the onset, dissemination and persistence of the inflammatory status in CRS [20]. Furthermore, the presence of bacterial biofilms may be associated with osteitis within the sinus bony frame [12].

The mechanism by which surgery improves the long-term outcomes and life quality in patients with chronic rhinosinusitis is not well understood. Probably, this could explain the fact that there is not yet an established consensus on how potential perioperative therapeutic targets such as mucosal healing and inflammatory changes should be managed (Jain et al., 2017) [23]. According to Lee et al., 2006 [24], early ESS was associated with high osteitis prevalence, greater endoscopic and imagistic severity scores and poor outcomes. Moreover, there is evidence of osteitis typically associated with nasal polyps, eosinophilia and recurrent CRS. Its pathogenesis remains unclear due to the lack of animal models [24,25]. Thus, with the development of new proper murine experimental models of osteitis after ESS, it becomes a necessity and, furthermore, an actual challenge.

This research follows a pilot study we conducted at the UMF Biobase ‘Iuliu-Hatieganu’, Cluj-Napoca, Romania, in February–September 2021. The main objective was to demonstrate the presence of neo-osteogenesis tissue associated with nasal endoscopic surgery by reproducing the intervention in an experimental murine model of induction of a mechanical nasal lesion. The results we obtained were similar to the existing data in the literature regarding the presence of neo-osteogenesis and mucosal inflammation associated with ESS. Inclusion criteria for osteitis, in different stages, were reported in 35.3% of the subjects. We identified inflammatory mucosal changes in 64.7% of the models. Once the association between endoscopic surgery and inflammation and neo-osteogenesis is demonstrated, the relevance of the current experiment comes from its aim to deepen the knowledge of the roles of inflammation and ossification in chronic rhinosinusitis (CRS).

To investigate the specific role of sino-nasal inflammation in bone remodeling associated with rhinosinusitis and the relationship between mucosal denudation and subsequent subjacent osteitis, we conducted our experimental models following the model proposed by Joo et al., 2019 [26]. Joo et al. describe the first experimental model on wound healing of small animals featuring exposed bone and neo-osteogenesis. Our research is the first that describes neo-osteogenetic changes at the level of the nasal turbinates following surgical injury. It has also demonstrated a statistically significant correlation between the mucosal inflammation score and the associated osteitis score. The more pronounced the inflammation at the level of the mucosa, the higher the osteitis score in the underlying bone. The reduction of inflammation at the level of the mucosa and faster epithelization—the effect of cryotherapy—are followed by a marked decrease in osteitis. Moreover, this is the first experimental study describing the presence of mature bone appearance with a similar incidence as the one with which the osteitis decreases.

Even though “true osteitis” with inflammatory infiltrate within the bone was not recognized in different research, osteitis appears to be a process of neo-osteogenesis and bone remodeling rather than bony inflammation [27]. Additionally, osteitis was described as exudate, inflammation and bony synechia [26]. In our research, we consider osteitis in CRS, any neo-osteogenesis process, bone resorption and bone remodeling.

Osteitis is a pathological dynamic process, and the mechanisms of formation and resorption that underpin it need to be studied further. Endoscopic surgeries’ poor outcomes may be due to the incomplete removal of the inflammatory bone, which perpetuates the regional inflammation and the consequent bone remodeling process [28]. Although there is evidence of bone inflammation in CRS, osteitis does not appear in all patients with chronic rhinosinusitis [20]. To evaluate the correlation between osteitis and inflammation, it may represent a solid argument for the involvement of the bone in the perpetuation of the inflammation within the mucosa. Our histopathological features and the subsequent statistical analysis demonstrated a relevant correlation between the inflammation of bone tissue and mucosal inflammation. As a result of our study, we suggest that the rapid restoration of epithelial integrity can significantly contribute to the reduction of osteitis. A challenge to define the ultimate standard in treatment, especially in recalcitrant CRS, still remains of great interest and should address the involvement of bone remodeling in chronic rhinosinusitis development.

The presence of mucosal and bony inflammation criteria, with a significantly higher incidence in control nasal fossae, clearly demonstrates the positive effect of low-pressure cryotherapy on surgery-induced mucosal and bony inflammation. The mucosal inflammation was found in 70.2% of the control vs. 29.8% of the experimental nasal fossae. Osteitis was highlighted in 76.7% of the control fossae vs. 23.3% of the cryo-treated fossae. In regard to the safety of the method, besides the subsequent epistaxis revealed in all specimens after the low-pressure liquid nitrogen application, no other adverse events were reported. The extended follow-up of 80 days, in comparison with 28–30 days of other research on inflammation, osteogenesis and bony maturation ([26] Joo et al., 2019; [20] de Campos et al., 2015), brought new insights into the osteitis process, such as the appearance of the mature lamellar bone after 60 days after induction. At 14 and 30 days after induction, osteitis was present in similar percentages, and after 80 days follow-up, bone remodeling decreased as the incidence by the same percentage with which mature lamellar bone deposition was identified, giving new insights on osteogenesis phases, resorption, new bone production and its maturation.

On the other hand, the usefulness of producing chronic inflammation and subsequent neo-osteogenesis similar to endoscopic sinus surgery by simple mechanical injury definitely represents a step forward in regard to similar experiments.

Compared to other previous experiments in the literature, such as Rosen needle injury [29] or the exposure to different biological agents such as Aspergillus Fumigatus [26], Pseudomonas Aeruginosa [30], staphylococcal and streptococcal toxoid [20], our study’s protocol complexity and technical difficulty are of low difficulty grade and high safety profile, with minimal stress on the animals.

Despite the existing literature’s rabbit and mouse models [2,11,25], we developed a murine model on rats because of the effortless handling, greater resistance, decreased costs and breed availability. We identified the impossibility of quantifying the degree of bone mineralization due to the embedded procedures as possible limitations of our research. To overcome this situation, specific expensive embedded procedures can be used. Additionally, other histological staining, such as Alizarin Red S and Polarized light microscopy, besides optic microscopy, were used to evaluate the histomorphometry of bone structure consistent with bone remodeling and to assess the orientation of collagen fibers, which could have brought more detailed and superior results [13,22].

Our research clearly demonstrates the presence of inflammation and osteitis after surgical injury and the correlation between the severity of inflammation and the severity of neo-osteogenesis. In the osteitis first grade, inflammation first grade is predominant and statistically significant. In osteitis second grade, inflammation second grade is the most frequent, and there are observable trends that higher inflammation grades are more prevalent in osteitis grade three.

We studied other possible therapeutic targets against osteitis and recurrent CRS by demonstrating the beneficial effects of low-pressure spray cryotherapy in reducing inflammation and bone remodeling. Specifically, the control nasal fossae were more prone to inflammation, while the control and cryo-treated nasal fossae showed a reduced prevalence of inflammation across the three analysed batches.

## 5. Conclusions

In our experimental murine model, we demonstrated that simple mechanical injury of the nasal mucosa may induce mucosal inflammation and bony osteitis changes, similar to surgical injury.

In our murine experimental model, we identified the presence of mucosal and bony inflammation criteria, which affected both the control and the cryo-treated side, with a significantly higher incidence in the control nasal fossae. The observed changes in terms of inflammation and bone remodeling have a zonal character, with a predilection for the anterior-most regions. Therefore, inflammation and osteitis were present over time in a statistically significant manner with a direct correlation between the two features.

Due to the prevalence of the histopathological features in control nasal fossae compared to the cryo-treated side and their statistical results, we can support the effectiveness of low-pressure cryotherapy in reducing the severity of mucosal inflammation and osteitis.

The extended 80-day follow-up offers significant new data on mature lamellar bone deposition and sustains the idea of bone remodeling and its stages.

This animal model could provide new insights into surgically induced osteitis, offering a better understanding and representing a basis for the development of more effective surgery and postoperative care, particularly for the difficult-to-treat CRS cases. Although cryotherapy seems safe and effective in previous investigations, further studies with validated values and controlled populations are certainly justified and should be encouraged.

## Figures and Tables

**Table 1:** Semiquantitative histopathological grading criteria after De Campos et al. [20].

	Grade	Histopathological Description
Mucosal Inflammation	0	no inflammation
	1	mild inflammation, presence of few mucosal inflammatory cells
	2	moderate inflammation, presence of diffuse inflammatory cells within the mucosa
	3	intense inflammation, presence of diffuse inflammatory infiltrate and epithelial injury, abnormal mucosal and submucosal architecture
Bone changes	0	no inflammation
	1	mild inflammation, with mild periosteal thickening
	2	moderate inflammation, presence of moderate periosteal thickening and osteoblastic rimming along the new bone
	3	intense inflammation, presence of noticeable periosteal thickening, osteoblastic rimming and non-mineralized osteoid

**Table 2:** Descriptive values on inflammation distribution by grades in all three batches.

			Batches			Total
			Batch Analysed at 14 Days	Batch Analysed at 30 Days	Batch Analysed at 80 Days	
Grades of inflammation	Inflammation first grade	Count	11	7	8	26
		% within Grades of inflammation	42.3%	26.9%	30.8%	100.0%
		% within Batches	57.9%	36.8%	42.1%	45.6%
		% of Total	19.3%	12.3%	14.0%	45.6%
	Inflammation second grade	Count	4	8	8	20
		% within Grades of inflammation	20.0%	40.0%	40.0%	100.0%
		% within Batches	21.1%	42.1%	42.1%	35.1%
		% of Total	7.0%	14.0%	14.0%	35.1%
	Inflammation third grade	Count	4	4	3	11
		% within Grades of inflammation	36.4%	36.4%	27.3%	100.0%
		% within Batches	21.1%	21.1%	15.8%	19.3%
		% of Total	7.0%	7.0%	5.3%	19.3%
Total		Count	19	19	19	57
		% within Grades of inflammation	33.3%	33.3%	33.3%	100.0%
		% within Batches	100.0%	100.0%	100.0%	100.0%
		% of Total	33.3%	33.3%	33.3%	100.0%

**Table 3:** Descriptive values on osteitis distribution by grades in all three batches.

			Batches			Total
			Batch Analysed at 14 Days	Batch Analysed at 30 Days	Batch Analysed at 80 Days	
Grades of osteitis	Osteitis first grade	Count	7	4	3	14
		% within Grades of osteitis	50.0%	28.6%	21.4%	100.0%
		% within Batches	41.2%	25.0%	30.0%	32.6%
		% of Total	16.3%	9.3%	7.0%	32.6%
	Osteitis second grade	Count	5	9	6	20
		% within Grades of osteitis	25.0%	45.0%	30.0%	100.0%
		% within Batches	29.4%	56.3%	60.0%	46.5%
		% of Total	11.6%	20.9%	14.0%	46.5%
	Osteitis third grade	Count	5	3	1	9
		% within Grades of osteitis	55.6%	33.3%	11.1%	100.0%
		% within Batches	29.4%	18.8%	10.0%	20.9%
		% of Total	11.6%	7.0%	2.3%	20.9%
Total		Count	17	16	10	43
		% within Grades of osteitis	39.5%	37.2%	23.3%	100.0%
		% within Batches	100.0%	100.0%	100.0%	100.0%
		% of Total	39.5%	37.2%	23.3%	100.0%

**Table 4:** Description of the correlation between the grade of inflammation and the grade of osteitis.

			Grades of Inflammation			Total
			Inflammation First Grade	Inflammation Second Grade	Inflammation Third Grade	
Grades of osteitis	Osteitis first grade	Count	11	2	1	14
		% within Grades of osteitis	78.6%	14.3%	7.1%	100.0%
		% within Grades of inflammation	78.6%	11.1%	9.1%	32.6%
		% of Total	25.6%	4.7%	2.3%	32.6%
		Adjusted Residual	4.5	−2.5	−1.9	
		Probability values	0.000	0.011	0.054	
	Osteitis second grade	Count	3	11	6	20
		% within Grades of osteitis	15.0%	55.0%	30.0%	100.0%
		% within Grades of inflammation	21.4%	61.1%	54.5%	46.5%
		% of Total	7.0%	25.6%	14.0%	46.5%
		Adjusted Residual	−2.3	1.6	0.6	
		Probability values	0.022	0.103	0.536	
	Osteitis third grade	Count	0	5	4	9
		% within Grades of osteitis	0.0%	55.6%	44.4%	100.0%
		% within Grades of inflammation	0.0%	27.8%	36.4%	20.9%
		% of Total	0.0%	11.6%	9.3%	20.9%
		Adjusted Residual	−2.3	0.9	1.5	
		Probability values	0.019	0.349	0.145	
Total		Count	14	18	11	43
		% within Grades of osteitis	32.6%	41.9%	25.6%	100.0%
		% within Grades of inflammation	100.0%	100.0%	100.0%	100.0%
		% of Total	32.6%	41.9%	25.6%	100.0%

**Table 5:** Descriptive data on inflammation distribution showing the predilection for the control nasal fossae.

			Batches			Total
			Batch Analysed at 14 Days	Batch Analysed at 30 Days	Batch Analysed at 80 Days	
Localization of inflammation	Control nasal fossae	Count	13	13	14	40
		% within Localization of inflammation	32.5%	32.5%	35.0%	100.0%
		% within Batches	68.4%	68.4%	73.7%	70.2%
		% of Total	22.8%	22.8%	24.6%	70.2%
		Adjusted Residual	−0.2	−0.2	0.4	
		Probability values	0.838	0.838	0.682	
	Cryo-treated nasal fossae	Count	6	6	5	17
		% within Localization of inflammation	35.3%	35.3%	29.4%	100.0%
		% within Batches	31.6%	31.6%	26.3%	29.8%
		% of Total	10.5%	10.5%	8.8%	29.8%
		Adjusted Residual	0.2	0.2	−0.4	
		Probability values	0.838	0.838	0.682	
Total		Count	19	19	19	57
		% within Localization of inflammation	33.3%	33.3%	33.3%	100.0%
		% within Batches	100.0%	100.0%	100.0%	100.0%
		% of Total	33.3%	33.3%	33.3%	100.0%

**Table 6:** Descriptive data on osteitis distribution, showing the predilection for the control side.

			Batches			Total
			Batch Analysed at 14 Days	Batch Analysed at 30 Days	Batch Analysed at 80 Days	
Localization of osteitis	Control nasal fossae	Count	12	12	9	33
		% within Localization of osteitis	36.4%	36.4%	27.3%	100.0%
		% within Batches	70.6%	75.0%	90.0%	76.7%
		% of Total	27.9%	27.9%	20.9%	76.7%
		Adjusted Residual	−0.8	−0.2	1.1	
		Probability values	0.440	0.835	0.257	
	Cryo-treated nasal fossae	Count	5	4	1	10
		% within Localization of osteitis	50.0%	40.0%	10.0%	100.0%
		% within Batches	29.4%	25.0%	10.0%	23.3%
		% of Total	11.6%	9.3%	2.3%	23.3%
		Adjusted Residual	0.8	0.2	−1.1	
		Probability values	0.440	0.835	0.257	
Total		Count	17	16	10	43
		% within Localization of osteitis	39.5%	37.2%	23.3%	100.0%
		% within Batches	100.0%	100.0%	100.0%	100.0%
		% of Total	39.5%	37.2%	23.3%	100.0%

## Data Availability

The data presented in this study are available on request from the corresponding author.
